# The first report of *RPSA *polymorphisms, also called 37/67 kDa LRP/LR gene, in sporadic Creutzfeldt-Jakob disease (CJD)

**DOI:** 10.1186/1471-2350-12-108

**Published:** 2011-08-13

**Authors:** Jisuk Yun, Hyoung-Tae Jin, Yun-Jung Lee, Eun-Kyoung Choi, Richard I Carp, Byung-Hoon Jeong, Yong-Sun Kim

**Affiliations:** 1 Ilsong Institute of Life Science, Hallym University, 1605-4 Gwanyang-dong Dongan-gu, Anyang, Gyeonggi-Do 431-060, Republic of Korea; 2 New York State Institute for Basic Research in Developmental Disabilities, 1050 Forest Hill Road, Staten Island, NY 10314, USA

## Abstract

**Background:**

Although polymorphisms of *PRNP*, the gene encoding prion protein, are known as a determinant affecting prion disease susceptibility, other genes also influence prion incubation time. This finding offers the opportunity to identify other genetic or environmental factor (s) modulating susceptibility to prion disease. Ribosomal protein SA (*RPSA*), also called 37 kDa laminin receptor precursor (LRP)/67 kDa laminin receptor (LR), acts as a receptor for laminin, viruses and prion proteins. The binding/internalization of prion protein is dependent for LRP/LR.

**Methods:**

To identify other susceptibility genes involved in prion disease, we performed genetic analysis of *RPSA*. For this case-control study, we included 180 sporadic Creutzfeldt-Jakob disease (CJD) patients and 189 healthy Koreans. We investigated genotype and allele frequencies of polymorphism on *RPSA *by direct sequencing or restriction fragment length polymorphism (RFLP) analysis.

**Results:**

We observed four single nucleotide polymorphisms (SNPs), including -8T>C (rs1803893) in the 5'-untranslated region (UTR) of exon 2, 134-32C>T (rs3772138) in the intron, 519G>A (rs2269350) in the intron and 793+58C>T (rs2723) in the intron on the *RPSA*. The 519G>A (at codon 173) is located in the direct PrP binding site. The genotypes and allele frequencies of the *RPSA *polymorphisms showed no significant differences between the controls and sporadic CJD patients.

**Conclusion:**

These results suggest that these *RPSA *polymorphisms have no direct influence on the susceptibility to sporadic CJD. This was the first genetic association study of the polymorphisms of *RPSA *gene with sporadic CJD.

## Background

Human prion diseases are fatal transmissible neurodegenerative disorders with accumulation of an abnormal infectious isoform (PrP^Sc^) in central nervous system and spleen. Approximately 85% of Creutzfeldt-Jakob disease (CJD) is sporadic CJD that differs from generically linked variant CJD in its relatively late age incidence and short duration of illness [1,2]. It is known that the conformational conversion of naturally expressed cellular prion protein (PrP^C^) to PrP^Sc ^is the key event in the pathogenesis of prion disease.

It is known that homozygous codon 129 of human prion protein gene (*PRNP*) increases the risk of CJD. Although about 40% of the British and about 95% of the Korean have this genotype, the incidences of sporadic CJD are similar [3-5]. This epidemiological data suggests that allelic frequencies of *PRNP *codon 129 are unlikely to be the sole genetic factor for sporadic CJD incidence [3]. Inbred mouse lines encoding the same *PRNP *genotype differed significantly in incubation time [6,7]. In addition, new quantitative trait loci (QTL) that affect prion disease incubation time have been detected [7-9]. These results make it possible to study other candidate loci and factors that play a role in the onset time of prion disease.

The 37 kDa laminin receptor precursor (LRP)/67 kDa laminin receptor (LR) is a multifunctional protein which acts as a receptor for laminin and is involved in organization of basement membrane [10]. LRP forms its mature LR by unknown mechanism involving post-translational fatty acid acylation [11,12]. LRP exists on the cell surface and LR is embedded in cell membrane [11]. The open reading frame (ORF) of the human LRP/LR gene, also called ribosomal protein SA (*RPSA*), is 888 bp in length, codes a protein of 295 amino acids and is found on chromosome 3p22.2. The amino acid sequence of *RPSA *shares high homology in mammals [10]. LRP/LR also acts as a receptor for many components; carbohydrates, elastin, green tea catching epigallocatechin-3-gallate (EGCG), various viruses, bacteria and prion protein [11,12]. LRP/LR can be a factor in pathological processes. In cancer, LRP/LR is upregulated and its overexpression is associated with tumor cell metastasis and attack [11,12]. In prion disease, LRP/LR acts as a receptor for PrP^Sc ^as well as a receptor or co-receptor for PrP^c ^[11-15]. PrP^c ^is co-localized with LRP/LR on the surface of mammalian cells [13]. Enhanced LRP/LR level was only found in systems converting PrP^c ^to PrP^Sc ^and in organs accumulating PrP^Sc ^such as brain, spleen and pancreas in scrapie-infected mice and hamsters [15]. Bovine prion protein (PrP^BSE^) after oral infection is internalized by human enterocytes via a specific LRP/LR-dependent process [16]. LRP/LR is essential for the propagation and accumulation of PrP^Sc ^in scrapie-infected cells, and its expression is related to the degree of PrP^Sc ^propagation [17,18]. Therefore, it is possible that PrP-LRP-LR interaction is related to pathogenesis of prion disease. LRP/LR also has potential as an alternative therapeutic strategy that aims at LRP interactions [19,20]. Recent studies showed that inactivation of LRP/LR interferes with either PrP^Sc ^propagation or PrP^Sc ^accumulation, leading to a prolonged pre-clinical phase and incubation times in scrapie-infected mice [21,22]. Catechin EGCG, a component in green tea, represents anti-allergic, anti-tumor and anti-obesity actions via binding to LRP/LR [23-25]. EGCG treatment interferes with the formation of PrP^Sc ^in scrapie-infected cells and with the stress-protective effect of PrP^c ^in uninfected cells [26].

Although PrP-LRP-LR interaction is important for prion disease, a relationship between *RPSA *polymorphism and prion disease has not been reported. Therefore, we investigated polymorphisms of human *RPSA*, and studied whether these polymorphisms are related to susceptibility to sporadic CJD.

## Methods

### Subjects

This study included 180 sporadic CJD patients (93 male and 87 female; mean age at disease onset 61.4 ± 12.0) and 189 healthy controls (89 male and 100 female; mean age at blood collection 71.7 ± 8.9) who were all unrelated and from an ethnically homogenous Korean population (Table 1). Blood samples were collected from May 1996 to April 2006.

**Table 1:** General characteristics of healthy controls and sporadic CJD patients

	healthy controls (n = 189)	Sporadic CJD patients (n = 180)	*P*-value
Gender			
Male, n (%)	89 (47.1%)	93 (51. 7%)	0.379
Female, n (%)	100 (52.9%)	87 (48.3%)	
Mean age at disease onset (yrs)	-	61.4 ± 12.0	<0.01
Mean age at blood collection (yrs)	71.7 ± 8.9	-	

Data are mean ± S.D. or percentage

The clinical criteria for sporadic CJD patients were established according to the WHO diagnostic criteria for definite or probable CJD [27]. In brief, definite CJDs were neuropathologically confirmed and/or immunochemically detected by presence of PrP^Sc ^in the brain. Probable CJDs were diagnosed by presence of progressive dementia, a typical electroencephalography (EEG), and/or a positive 14-3-3 protein in CSF and a clinical duration leading to death in <2 years and at least two of the following: myoclonus, visual or cerebellar disturbance, pyramidal, extrapyramidal dysfunction, akinetic mutism. Patients with mutation in the *PRNP *were excluded from this study. 12 definite sporadic CJD and 168 probable sporadic CJD cases were enrolled in our study.

All healthy control subjects participated voluntarily during routine checkups at the Chuncheon Sacred Heart Hospital and Hallym University Sacred Heart Hospital. The purpose of the study was carefully explained to all study participants or their representatives and their informed consents were obtained. The study complied with the Guidelines for Genome/Genetic Research issued by the Korean government and was approved by the Ethical Committee of Hallym University Sacred Heart Hospital, Anyang, Korea.

### Genetic analysis

Whole blood samples were drawn into ethylenediaminetetraacetate (EDTA)-treated tubes and were kept frozen at -70°C until analysis. Genomic DNA was extracted from whole blood using the QIAamp^® ^DNA blood Mini Kit (QIAGEN, Valencia, CA, USA) according to the manufacturer's instruction. For the screening polymorphisms in the LRP/LR, polymerase chain reaction (PCR) was performed with forward primer and reverse primer (Table 2). A 50 μL reaction mixture containing 10 μL of genomic DNA, 1 μL each of forward and reverse primers, 4 μL of 2.5 mM dNTP mixture, 5 μL of 10X Taq DNA polymerase buffer (Promega, Madison, WI., USA), 0.5 μL of 2.5units Taq DNA polymerase (Promega, Madison, WI., USA) and 23.5 μL of sterile deionized water was placed in PTC-200 Peltier Thermal Cycler (MJ Research, Reno, NV, USA). PCR reactions were thermally cycled with an initial denaturation at 90°C for 3 minutes, followed by 35 cycles of 95°C for 30 seconds, 57°C for 30 seconds and 72°C for 1.5 minutes and then a final extension at 72°C for 4 minutes. Each PCR product was run on a 1% agarose gel stained with ethidium bromide (EtBr), and then was purified by QIAquick^® ^Gel Extraction Kit (QIAGEN, Valencia, CA., USA). The PCR products were directly sequenced with an ABI 3730 Capillary Electrophoresis Sequencer (Applied Biosystems, Foster City, CA, USA).

**Table 2:** PCR primers used to screen the *RPSA*

Primer identity	Primer sequences	Nucleotide position	Product size
*RPSA *1	A: GAT GTG CGC TGT TCC GTA AT B: TCA TGT TCA TGA CCC AAC CC	1026-1601	576 bp
*RPSA *2	A: GGA AAG AGT GGC AGA AAG CC B: TGC TGG GAT TAC AGG CGT AG	1866-2431	566 bp
*RPSA *3	A: CCA GTG CCC AGA AGT GCT TA B: TTC ATC GGC CAG TCA GTA GC	4252-4681	430 bp
*RPSA *4	A: GCT TGC TGT TTG GGT TTG AC B: TTC CTT ACC CCA CTC CCA AC	5171-5799	629 bp
*RPSA *5	A: AAG CAA AAC TTG TCA GTC CCT G B: CAA CCA TTT TTC CAT GCT GC	5421-5990	570 bp

'A' indicates the forward primer, and 'B' indicates the reverse primer.

Alleles of *RPSA *2, *RPSA *4 and *RPSA *5 were determined by PCR restriction fragment length polymorphism (RFLP). Restriction sites were searched using Webcutter 2.0 http://rna.lundberg.gu.se/cutter2. Restriction digests (enzymes supplied by Fermentus) were performed in a 20 μL reaction mixture containing 10 μL of PCR reaction mixture, 2 μL of 10× buffer B, 0.5 μL of TasΙ and 7.5 μL of sterile deionized water was digested at 65°C for 4 hours. Digested PCR products were electrophoresed on a 2% agarose gel and visualized with EtBr staining under UV light.

### Statistical analysis

Statistical data were carried out using Statistical Analysis Software (SAS), version 8.2 (SAS Institute Inc., Cary, NC., USA). The χ^2 ^test was used to assess differences between categorical variables. Difference between the two groups regarding genotype frequencies were determined by Fisher's exact test, and comparisons of allele frequencies were assessed by χ^2^-test. Student's t-test was used to analyze differences between the normal population and sporadic CJD patients by age. Hardy-Weinberg Equilibrium test and haplotype analysis were performed by use of the SNPAnalyzer 1.2A http://snp.istech21.com/snpanalyzer/1.2A/. Linkage disequilibrium (LD) between paired polymorphisms was tested.

## Results

To identify an association between *RPSA *polymorphisms and sporadic CJD, we screened nucleotide variations in *RPSA *using direct gene sequence or RFLP analysis. We observed four single nucleotide polymorphisms (SNPs), including -8T>C (rs1803893) in the 5'-untranslated region (UTR) of exon 2, 134-32C>T (rs3772138) in the intron, 519G>A (rs2269350) in exon 5 and 793+58C>T (rs2723) in the intron. The genotype frequencies of all SNPs followed Hardy-Weinberg equilibrium except for *RPSA *793+58C>T in controls (data not shown).

We analyzed the extent of LD between the polymorphisms. Four SNPs identified in the *RPSA *were strongly linked together with *D*' values 0.94 - 1.00 (Table 3). Because four *RPSA *polymorphisms are in strong LD, we analyzed the genotype frequency of *RPSA *5'-UTR -8T>C using direct sequencing, whereas frequencies of other genes were determined using RFLP analysis. As shown in Table 4, there was no significant difference in the genotype distribution of the *RPSA *polymorphisms between controls and sporadic CJD groups. Allele frequencies of *RPSA *polymorphisms also showed no differences between these two groups. In addition, the genetic variations of these polymorphisms were equally frequent by gender (data not shown).

**Table 3:** *D' *Values for Linkage Disequilibrium (LD) between *RPSA *polymorphisms

	5'-UTR -8T>C	134-32C>T	519G>A	793+58C>T
5'-UTR -8T>C	-	1.00	1.00	0.94
134-32C>T	-	-	1.00	0.96
519G>A	-	-	-	1.00
793+58C>T	-	-	-	-

**Table 4:** Genotype and allele frequencies of *RPSA *polymorphisms in controls and sporadic CJD patients

	5'-UTR -8T>C		134-32C>T		519G>A		793+58C>T	
				
	Controls (n = 189)	Sporadic CJDs (n = 180)	Controls (n = 50)	Sporadic CJDs (n = 50)	Controls (n = 50)	Sporadic CJDs (n = 50)	Controls (n = 50)	Sporadic CJDs (n = 50)
Genotype								
1/1	91 (48.2%)	87 (48.3%)	33 (66.0%)	33 (66.0%)	34 (68.0%)	33 (66.0%)	28 (56.0%)	24 (48.0%)
1/2	77 (40.7%)	78 (43.3%)	15 (30.0%)	15 (30.0%)	14 (28.0%)	15 (30.0%)	12 (24.0%)	22 (44.0%)
2/2	21 (11.1%)	15 (8.4%)	2 (4.0%)	2 (4.0%)	2 (4.0%)	2 (4.0%)	10 (20.0%)	4 (8.0%)
*P-*value	0.646		1.000		1.000		0.058	

Allele								
1	279 (0.70)	252 (0.70)	81 (0.81)	81 (0.81)	82 (0.82)	81 (0.81)	68 (0.68)	70 (0.70)
2	119 (0.30)	108 (0.30)	19 (0.19)	19 (0.19)	18 (0.18)	19 (0.19)	32 (0.32)	30 (0.30)
*P-*value	0.976		1.000		0.856		0.760	

For columns '1' refers to the more common genotype or allele and '2' to the less common genotype or allele of each polymorphism.

As shown in Table 5, five different haplotypes exist in the *RPSA *polymorphisms: TCGT, CTAC, TCGC, CCGC and CTGT. Among the five haplotypes, the haplotype TCGT was observed more frequently (67% for controls; 69% for sporadic CJD patients); the second major haplotype was CTAC (18% for controls; 19% for sporadic CJD patients). The haplotype CTGT was less frequently observed in both controls and sporadic CJD patients (1% for both controls and sporadic CJD patients). Analysis of the haplotype frequencies of *RPSA *polymorphisms showed no significance between controls and sporadic CJD patients.

**Table 5:** Haplotype frequency of *RPSA *polymorphisms in controls and sporadic CJD patients

Haplotype	Controls (n = 50)	Sporadic CJDs (n = 50)	*P*-value
TCGT	67.0%	68.9%	-
CTAC	18.0%	19.0%	0.943
TCGC	9.0%	6.1%	0.574
CCGC	5.0%	4.9%	0.714
CTGT	1.0%	1.1%	0.983

In order to identify whether the *RPSA *polymorphism affects susceptibility to sporadic CJD, we tested for LD with *PRNP *codon 129 polymorphism. Because four *RPSA *polymorphisms have strong LD, we only tested *RPSA *1 polymorphism. The *RPSA *5'-UTR - 8T>C polymorphism is in low LD with both *PRNP *codon 129 polymorphism and 219 polymorphism (*D*' = 0.497 and *D*' = 0.110, respectively).

To identify the combined effects of *RPSA *and *PRNP *on susceptibility to prion disease, *RPSA *5'-UTR -8T>C genotype data was stratified with *PRNP *codon 129 and with codon 219 genotypes (Table 6). We found no significant differences in the genotype and allele frequencies of *RPSA *5'-UTR -8T>C polymorphism according to the *PRNP *codon 129 status. Similar results were seen when stratified with *PRNP *codon 219.

**Table 6:** Genotype and allele frequencies of *RPSA *5-UTR -8T>C polymorphism according to the *PRNP *codon 129 or 219 status

*PRNP *129	Healthy controls (n = 189)			Sporadic CJD patients (n = 180)			*P-*value^1^	*P-*value^2^
				
	MM (n = 180)	MV (n = 9)	VV (n = 0)	MM (n = 180)	MV (n = 0)	VV (n = 0)		
Genotype frequency, n (%)								
TT	84 (46.7)	7 (7.8)	-	87 (48.3)	-	-		
TC	75 (41.7)	2 (2.2)	-	78 (43.3)	-	-	0.233	0.570
CC	21 (11.6)	0 (0)	-	15 (8.3)	-	-		
Allele frequency								
T	0.68	0.89	-	0.70	-	-	0.057	0.469
C	0.32	0.11	-	0.30	-	-		

***PRNP *219**	**Healthy controls (n = 189)**			**Sporadic CJD patients (n = 180)**			***P-*value^3^**	***P-*value^4^**
				
	**EE (n = 173)**	**EK (n = 16)**	**KK (n = 0)**	**EE (n = 180)**	**EK (n = 0)**	**KK (n = 0)**		

Genotype frequency, n (%)								
TT	86 (49.7)	5 (31.3)	-	87 (48.3)	-	-		
TC	68 (39.3)	9 (56.3)	-	78 (43.3)	-	-	0.338	0.600
CC	19 (11.0)	2 (12.4)	-	15 (8.3)	-	-		
Allele frequency								
T	0.69	0.59	-	0.70	-	-	0.244	0.854
C	0.31	0.41	-	0.30	-	-		

^1^Difference in healthy controls with MM and healthy controls with MV.

^2^Difference in healthy controls with MM and sporadic CJD patients with MM.

^3^Difference in healthy controls with EE and healthy controls with EK.

^4^Difference in healthy controls with EE and sporadic CJD patients with EK.

## Discussion

In order to find association of *RPSA *with sporadic CJD, we screened sequence of the *RPSA *in all available samples of sporadic CJD patients from Korean populations and investigated SNPs in the LRP/LR gene. In this study, we identified four polymorphisms including 519G>A (at codon 173) in the coding region and three SNPs located in 5'-UTR and intron on the *RPSA*. By two-hybrid system in the yeast and cell-binding assay, two PrP-LRP-LR interaction domains on *PRNP *were identified: a direct binding domain amino acids 144-179, termed PrPLRPbd1 and an indirect heparin sulfate proteoglycan (HSPG)-dependent LRP binding domain amino acids 53-93, termed PrPLRPbd2 [28]. On *RPSA*, the direct PrP binding site is amino acids 161-180 and the heparin sulfate-dependent PrP-binding site is amino acids 205-229 [29]. The *RPSA *polymorphism at codon 173 is a synonymous SNP, which encodes leucine, and does not lead to substitution of amino acid. Nevertheless, it remains of interest, because the polymorphism at codon 173 is located in the direct PrP binding domain. Similar to our study, ovine *RPSA *polymorphisms in the regions related to PrP-LRP-LR interaction did not lead to a change in amino acid sequence [30]. Although the effects of this SNP on LRP/LR biology or prion biology are unknown, it is possible that the differences in combinations of amino acids may modify its specificity with other proteins affecting PrP-LRP-LR interaction, strengthening or weakening the species barrier [30]. The *RPSA *amino acid sequences associated with resistance barrier to scrapie infection have been identified at positions 241, 272 and 291 in goat [31], and at positions 241, 271 and 290 in sheep [30]. SNPs in UTR are known to have an effect on the expression or stability of genes. It is possible that the 5'-UTR -8T>C polymorphism may be responsible for genetic susceptibility to sporadic CJD.

In addition to differences in LRP/LR amino acid sequence, which affects the interaction with PrP^Sc^, differences in the production/expression of LRP/LR bioavailability at the cell surface and particular conformations might be regional leading to cellular differences in binding/accumulation of prion proteins; the result of these differences could lead to variable response to scrapie infection [30,32]. LRP is relatively abundant in developing brain tissue, and its expression is limited to a few neurons in cortex known to be especially sensitive to abnormal prion accumulation, and these cells rapidly degenerate during the early stages of CJD [32,33]. LR is expressed in most adult neurons, and a subset of glial cells and astrocytes, which can accumulate PrP^Sc ^in scrapie-infected hamsters [32]. PrP^C^-LRP-LR interaction, related to prion protein binding/accumulation, may take place at the cell soma and apical dendrites [32]. LRP is localized in the cytoplasm and in the nucleus, and LR can be present as a laminin-binding protein on the cell surface or in a free form in the extracellular matrix [11,30].

As shown in Table 4, although we found no significant difference in the genotype and allele frequencies of the *RPSA *polymorphisms between controls and sporadic CJD groups, *RPSA *793+58C>T showed a tendency to lower frequency of heterozygotes in sporadic CJD patients than those of controls (*P *= 0.058). Since the number of *RPSA *793+58C>T was much smaller (n = 50 for controls, n = 50 for sporadic CJD patients), further study on whether this result relates to our small sample sizes is required. In our study, the genotype and allele frequencies of *RPSA *polymorphisms were not influenced by gender. Lloyd et al. [7] and Stephenson et al. [8] reported that prion disease incubation time is not affected by sex. In contrast, the studies of Moreno et al. [9] showed that the survival time of female mice is shorter than those of male mice. The source of this sex effect could be hormonal factors, body size, fat composition, appetite and so on, acting at the end of the survival time [9].

One major haplotype (TCGT) is present in both controls and sporadic CJD patients with frequencies of 67-69% (Table 5). The additional four haplotypes were not significantly different in the two populations.

Some SNPs may act through LD with other mutations, even if it may not have direct influence on sporadic CJD susceptibility. From this point of view, we tested for LD between *RPSA *polymorphisms and *PRNP *codon 129. We found that *RPSA *polymorphisms had no effect on the susceptibility of 129MM individuals to sporadic CJD. To fully elucidate the association of the *RPSA *polymorphisms with sporadic CJD susceptibility, further studies are required using various populations because different linkage patterns can occur in different populations.

The *PRNP *associated resistance/susceptibility for prion disease is likely to coexist with other genes modulating its effect [30]. In previous studies, we found that sporadic CJD shows a susceptibility effect at codon 129 and 219 on *PRNP *in Koreans [4]. The *PRNP *polymorphism at codon 219 is unique to Asian populations, and *PRNP *219EK heterozygous genotype has a protective effect on sporadic CJD development [4]. In analysis stratified by *PRNP *codon 129 or 219 status, we found no significant difference in the genotype and allele frequencies of *RPSA *5'-UTR -8T>C polymorphism according to the *PRNP *codon 129 or 219 status (Table 6). These results are a function of the fact that all sporadic CJD patients had only *PRNP *129MM or 219EE genotype.

## Conclusions

Although *RPSA *alone is not a disease-modifying gene for sporadic CJD in Koreans, these SNPs might have an effect on the expression or susceptibility of *RPSA *gene and PrP^C^-LRP-LR interaction. In this case-control study, we included only Korean populations; therefore this result may not be generalized to other ethnicities. There are comparatively few Koreans diagnosed with sporadic CJD; this contributes to the limitation of the power analysis. Nevertheless, this study is important in that it forms the foundation of genetic association study on *RPSA *gene and sporadic CJD. Further genetic studies on whether variants in *RPSA *gene are associated with human prion disease should examine various ethnicities and larger numbers.

## Competing interests

The authors declare that they have no competing interests.

## Authors' contributions

JY, BHJ, and YSK designed the study. JY, HTJ, and YJL performed the genotyping. JY, EKC, RIC, BHJ, and YSK analyzed the data. JY and BHJ performed statistical analysis. JY, RIC, BHJ, and YSK wrote the paper. All authors read and approved the final manuscript.

## Pre-publication history

The pre-publication history for this paper can be accessed here:

http://www.biomedcentral.com/1471-2350/12/108/prepub
